# Questionnaire Translation, Adaptation, and Co‐Creation of the Swedish Version of the Fear of Pain Questionnaire Child Report Short Form

**DOI:** 10.1002/pne2.70011

**Published:** 2025-07-04

**Authors:** Malin Lanzinger, Sophie Jörgensen, Marcelo Rivano Fischer, Laura E. Simons, Jan Lexell

**Affiliations:** ^1^ Department of Health Sciences, Rehabilitation Medicine Research Group Lund University Lund Sweden; ^2^ Department of Neurosurgery and Pain Rehabilitation Skåne University Hospital Lund Sweden; ^3^ Department of Rehabilitation Medicine Skåne University Hospital Lund Sweden; ^4^ Department of Anesthesiology, Perioperative and Pain Medicine Stanford University School of Medicine Palo Alto California USA; ^5^ Department of Rehabilitation Medicine Ängelholm Hospital Ängelholm Sweden

**Keywords:** pain‐related fear, pediatric chronic pain, self‐report questionnaire, translations

## Abstract

Pain avoidant behavior is a predictive factor for reduced function in children with persistent pain. A model to explain this is the Fear‐Avoidance Model of Pain (FAM). In FAM pain‐related fear plays an important role in the development and maintenance of avoidant behavior. The Fear of Pain Questionnaire—Child Report Short Form (FOPQC‐SF) was developed to assess pain‐related fear in children 8 to 18 years old. Self‐report questionnaires must be translated and adapted to the language and context where they will be used. The aim of this study was therefore to develop a Swedish version of the FOPQC‐SF in collaboration with children. Translation and linguistic adaptation of the FOPQC‐SF was performed using the dual‐panel method in two steps. First, a bilingual panel created a first Swedish version of the questionnaire. This first version was then presented to a panel of five children without persistent pain and revised according to their feedback. Secondly, the translated and revised Swedish version of the FOPQC‐SF was used for individual cognitive interviews with six children with persistent pain. The bilingual panel found the FOPQC‐SF unproblematic to translate and consensus was easily achieved. Revisions were made regarding instructions, response options, item‐wording and layout. The children also found the questionnaire acceptable and relevant. In conclusion, we consider the Swedish version of the FOPQC‐SF to be a relevant and useful tool in research as well as in clinical practice to assess pain‐related fear. Psychometric testing will provide further information about the tool's clinical usefulness.

## 1 Introduction

Persistent pain in children and adolescents (further referred to as children) is a worldwide problem, widely unrecognized and often undertreated [1]. The prevalence varies depending on the target population, the pain definition used, and how pain is assessed [1]. A study including over 200 000 children aged 11, 13 and 15 years from 42 countries showed that 44% reported pain every week during the preceding 6 months [2]. A systematic review and metanalysis showed that approximately 20% of children and adolescents experience persistent pain, with a higher prevalence among girls than boys [3]. For some children with persistent pain the consequences become extensive and include school absence, physical inactivity, poor mental health and reduced quality of life [1, 4, 5, 6, 7], emphasizing the need for a biopsychosocial approach in pain assessment, treatment and rehabilitation [1, 8, 9].

Pain avoidant behavior has been identified to be a predictive factor for reduced physical, social and psychological function as well as quality of life. A widely used explanatory model is the “Fear‐Avoidance Model of Pain” (FAM) [10, 11, 12]. In FAM, pain‐related fear plays an important role in the development and maintenance of avoidant behavior, which leads to inactivity and disability. Pain‐related fear is an overarching complex phenomenon comprising different dimensions such as fear of reinjury, fear of movement, fear of physical activity, fear‐avoidance beliefs and fear of pain itself [13, 14]. Children rarely report fear of pain explicitly, instead they often show avoidance of activities and specific behaviors in different contexts. Therefore, there is a need for improved knowledge about pain‐related fear and how to address it in the assessment, treatment, and rehabilitation of children with persistent pain.

A self‐report questionnaire developed to assess pain‐related fear in children, 8 to 18 years, with persistent pain is the Fear of Pain Questionnaire—Child Report (FOPQC) [14]. To reduce the respondent burden, a short form (FOPQC‐SF) comprising 10 items derived from the original version has been developed and validated [15]. Both FOPQC and FOPQC‐SF are theoretically based on the FAM and are the most frequently used questionnaires to assess pain‐related fear in children with persistent pain.

Self‐report questionnaires must be translated and adapted to the language and context where they will be used. The most used method for translation is a forward‐backward translation. As this is often performed by professional translators, field experts, or healthcare professionals, there is a risk that the wording and comprehensibility of the questionnaire are not optimal for the target group and thereby affect the content validity. This risk increases when children are the target group since their comprehension of words and expressions may differ from adults. An alternative method for translation and adaptation is the dual‐panel method [16, 17]. Translations developed with this method have been shown to be preferred by target groups compared to versions developed through a conventional forward‐backward translation, and they have also been found psychometrically comparable [16, 18]. In addition, it has been suggested that the advantages of dual‐panel translations from the patients perspective may contribute to higher item response rates [16]. The dual‐panel method involves lay people in the translation process at an early stage and can be combined with cognitive interviews of persons from the target group [19, 20]. Thereby, the translation and adaptation of a questionnaire are optimized and the quality of the process to produce a conceptually valid questionnaire is ensured.

The aim of this study was to develop a Swedish version of the FOPQC‐SF in collaboration with children and adolescents (i.e., target group) using the dual‐panel method combined with cognitive interviews.

## 2 Materials and Methods

### 2.1 Study Design

**FIGURE 1:**
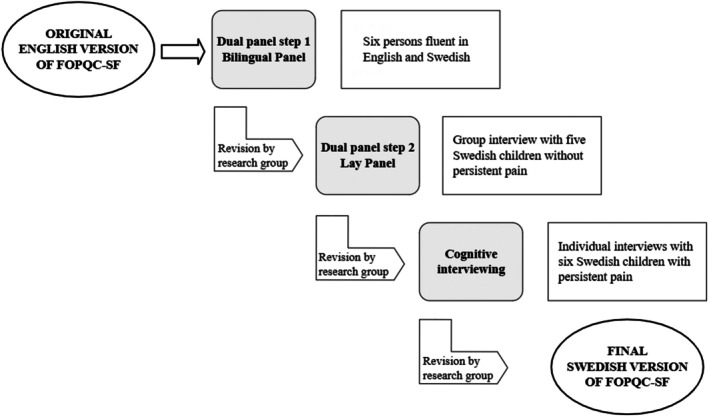
The translation and adaptation process based on the dual‐panel method and cognitive interviewing.

The FOPQC‐SF was translated and adapted by combining two methods: the dual‐panel method followed by cognitive interviewing. In Figure 1, the whole translation and adaptation process is summarized. After each step of the dual‐panel method and after cognitive interviewing, data from the interviews were analyzed and discussed, and thereafter decisions regarding revisions of the questionnaire were made. Questions to be answered throughout the process were: Do the respondents understand the instructions of the questionnaire? Are the respondents able to answer the questionnaire? Do the respondents understand the items? Do the respondents find the FOPQC‐SF relevant and usable?

The Swedish research group consisted of persons with clinical and academic expertise regarding the concept of pain‐related fear and self‐report questionnaires, as well as quantitative and qualitative interviewing and analysis. This expertise, combined with the involvement of the original developer of the FOPQC‐SF (Professor Laura E. Simons) in the process and the manuscript writing, was considered to secure the intention and concept of the translated Swedish version throughout the process.

### 2.2 The Fear of Pain Questionnaire—Child Report—Short Form (FOPQC‐SF)

The FOPQC‐SF is a self‐report questionnaire with 10 items derived from the FOPQC. The FOPQC [14] comprises 24 items distributed in two subscales: (i) pain‐related fear and (ii) avoidance of activity. For the short version, 10 items that best represent the content and 2‐factor structure of the original measure were selected [15]. Confirmative factor analyses supported the two‐subscale solution in the short version: pain‐related fear (4 items) and avoidance of activity (6 items) [15].

In the FOPQC‐SF, items are rated on a 5‐point Likert scale: 0 = strongly disagree; 1 = disagree; 2 = unsure; 3 = agree; 4 = strongly agree. Item scores are then summed to derive a total score of 0 to 40 points. Greater scores indicate higher levels of pain‐related fear and avoidance. The FOPQC‐SF has shown strong internal consistency (Cronbach's Alpha 0.84 for total score) as well as strong test–retest reliability (Pearson's correlation 0.635 between baseline and 3‐month follow‐up), and there is evidence of responsiveness to change [15]. The original FOPQC‐SF is presented in Appendix S1.

### 2.3 Dual‐Panel Method

The translation and linguistic adaptation of the FOPQC‐SF was performed using the dual‐panel method in two steps [17]. In step 1, a bilingual panel created a first version of the questionnaire. In step 2, this first version was presented for and revised in collaboration with a panel of lay people.

#### 2.3.1 Bilingual Panel

The bilingual panel consisted of six persons, all fluent in Swedish and English. Four of them are part of the research group (M.L., S.J., M.R.F. and J.L.) with a strong theoretical understanding of the questionnaire and clinical rehabilitation experience from children and adults with persistent pain. One person has English as native language (C.B.) and one person is teaching English at the university level (E.K.).

The bilingual panel had a 3 h meeting to produce a first version of the Swedish FOPQC‐SF. They discussed the translation of instructions, the response options, and the items until consensus was achieved. The panel also worked with the developer of the original questionnaire (L.S.) and discussed specific items to ensure preservation of the conceptual meaning of FOPQC‐SF.

#### 2.3.2 Lay Panel

The Swedish version of FOPQC‐SF produced by the bilingual panel was then reviewed by a lay panel comprising three girls and two boys (aged 12 to 16 years without persistent pain). The revision of the Swedish FOPQC‐SF by the lay panel aimed to acknowledge if appropriate concepts were maintained and if individual items were comprehensible and acceptable in their content and wording. The five children have Swedish as their native language and were recruited from the network of the authors. The review took place at the local hospital, after school time, and lasted for 45 min.

The first author (M.L.) informed the lay panel of the questionnaire, its purpose and target group, led the group discussion and ensured that all participants were active in the discussion. The first author took active part by asking questions, clarifying uncertainties and guided the group to ensure that the discussion and suggestions were consistent with the purpose of the questionnaire. The children had access only to the Swedish version of the questionnaire and discussed the items regarding language and comprehension. If the children identified a need for change, they were asked for alternatives.

The lay panel interview was recorded and transcribed by the first author. The report was then used when the research group met to discuss the comments and suggestions from the lay panel. Linguistic changes were made, but only if the purpose and theoretical ground of the questionnaire were maintained. A new version of the questionnaire was finalized, which was used for the cognitive interviewing. Once again, the developer of the original questionnaire (L.S.) was engaged in this phase of the process.

### 2.4 Cognitive Interviewing

The cognitive interviewing was informed by the method of Willis [21]. Through cognitive interviews, the participants' understanding of the language and content of the questionnaire was examined. The cognitive interviews also contributed to an increased knowledge of the content and face validity of the questionnaire.

#### 2.4.1 Participants and Procedure

Six children, fluent in Swedish, were recruited from the Specialist clinic for pediatric persistent pain at Skåne University Hospital, Sweden. They were 12 to 17 years old, had Swedish as their native language, and had persistent limb pain, abdominal pain, or headache for at least 6 months; five of them had completed a pain rehabilitation program at the clinic. The selection of participants was strategic regarding age, gender, and pain location.

The cognitive interviews were conducted individually using a semi‐structured interview guide, and each interview lasted 25 to 50 min. One interview was carried out digitally, the other interviews took place in person at the hospital. Before the start of the interview the child was given the following information:

I'm interested in knowing what you are thinking when you are reading and answering the questionnaire and therefore, I would like you to think out loud – this makes it easier for me to understand how you understand what's written and how you choose to answer. I am not the one who developed the questionnaire so you will not make me upset if you think something is strange or difficult to understand. There are no right or wrong answers. So, do you have any questions before we start?

The child then answered the entire questionnaire and thought aloud when doing so. It was noted if and how there was any hesitation; this observation was then discussed later with the child during the interview. If the child did not understand a question this was responded to after completion of the entire questionnaire. After completion of the questionnaire, general and specific probes were used to obtain information on comprehension, language, relevance, usability and choices of response (i.e., retrospective probing). Appendix S2 gives an overview of the cognitive interview procedure with examples of probes.

All interviews were conducted by the first author (M.L.) who is a trained physiotherapist as well as a Cognitive Behavioral Therapy therapist with experience in conducting clinical exploratory interviews with children with persistent pain. The interviewer gained knowledge about cognitive interviewing by reading relevant literature, and by discussing with researchers who had previously used the method and generously shared their experiences and interview protocols.

#### 2.4.2 Data Analysis and Reporting

All interviews were recorded and transcribed verbatim. From the transcribed interviews, data consistent with the scope of the study, the theoretical construct of the questionnaire, and data indicating difficulties in comprehension and/or responding were extracted and summarized in a written report. This report was then used by the research group for further analysis. Our focus when extracting and analyzing data from the lay panel interview was to detect difficulties in linguistic comprehension. From the cognitive interviews the focus was the same but also included difficulties in responding to items. Decisions to revise or not were based on data from the interviews, the theoretical foundation of the questionnaire and our expertise in the field.

To structure the written report of the interviews, the Cognitive Interviewing Reporting Framework (CIRF) was used as a guide [22]. CIRF consists of a 10‐point checklist for reporting cognitive interviews aiming to improve the quality of reports.

### 2.5 Ethical Considerations

The study followed the Declaration of Helsinki and was approved by the Swedish Ethical Review Authority (number 2022‐03786‐01). All children and parents were given age appropriate written and verbal information about the study and thereafter gave their written informed consent to participate. For children under the age of 15 years written informed consent was given by one parent.

To minimize the burden for the child, the interviews were scheduled at times that suited them, and the number of interviews was kept at a minimum. The children were informed that participation was voluntary and that they had the right to withdraw without explaining why and without any consequences for their further treatment. No risks in participating in the project were identified; yet talking about a pain condition and answering questionnaires might sometimes raise discomfort such as emotional reactions. The participants were therefore informed that this could happen, and, if so, they would be offered support from health care; none of the children expressed any need of support.

## 3 Results

### 3.1 Bilingual Panel

The instruction and most of the items were found unproblematic to translate and consensus was easily achieved. For some of the items, as well as the wording among the response options, the meaning and intention had to be discussed and agreed on before translation. Regarding the intention and purpose of the response option “unsure”, the developer of the FOPQC‐SF (L.S.) was involved in the discussion. The intention of “unsure” was to be the option when the child cannot decide (“neither agree nor disagree”). The bilingual panel thereby identified a need for this response option to be specifically addressed by the lay panel to ensure that the children responded as intended. Another example of wordings that needed to be addressed by the lay panel was the expression “my heart beats fast or races.” We wanted to know how these wordings were interpreted by the children to ensure that the translation fulfilled the intention of the item, namely feeling anxious or stressed.

### 3.2 Lay Panel

Overall, the children thought that answering the questionnaire would be a good thing if they were to seek healthcare due to persistent pain, and they did not think that it would be difficult to answer the questionnaire. The response option “unsure” was identified as problematic also by the lay panel and could be interpreted in different ways. The lay panel had no suggestions on alternative wordings and the research group decided to keep “unsure” for further testing during the cognitive interviews. Further revisions made after the lay panel interview were to remove the word “body” from the first item (since this was implied) and add “to me” in item 2 (“When I feel pain, I am afraid that something terrible will happen *to me*”). “My heart beats fast or races” was interpreted as being afraid or nervous. The lay panel reflected that “beats fast or races” are two words with the same meaning and it was therefore decided to remove the word “races”. Other suggestions from the lay panel leading to revisions were to simplify the language to make it easier and more in line with how young people talk and write, and to make some changes in the layout to enhance readability.

### 3.3 Cognitive Interviewing

The presentation of the results from the cognitive interviews follows the structure of the FOPQC‐SF (instructions, response options and item 1–10) with the addition of perceived relevance and usability of the questionnaire. In the following, the results are presented with representative and illustrating citations. The final Swedish version of the FOPQC‐SF is presented in Appendix S3.

#### 3.3.1 Instructions

In the instruction to the FOPQC‐SF the respondents are asked about having pain for “a few hours or days”. The children did not recognize themselves in this timeframe since they had experienced pain for longer periods or shorter recurrent episodes of pain. It was therefore decided to change the instruction to “often or for a longer time” which includes a broader and less specified variation of persistent pain.

#### 3.3.2 Response Options

The middle response option (i.e., unsure) was problematic and interpreted differently. “Unsure” could mean anything from “both,” “sometimes,” “is a little bit true,” “in between” and “perhaps,” and was hard to relate to for the children. One child did not choose this option for any item. Considering these difficulties, it was decided to change the scale and only let the anchors be explained in writing (i.e., strongly disagree and strongly agree).

I don't know what unsure means. It is a bit confusing. (Child 5)

#### 3.3.3 Items

The interviews resulted in increased knowledge about how items were understood and responded to. Two items, 3 and 5, were reformulated after the cognitive interviews, whereas the other items were kept unchanged.

*Item 1. I can't do all the things normal people do because it's so easy to hurt my body*.

To get hurt was interpreted as a risk to hurt the body, which is the intention of the item and supports the reflections from the lay panel about the word “body” being implied. “Get hurt” could be related to the existing pain condition but to some children it meant something that happens suddenly and not related to the existing pain, like an ankle sprain. They also reflected that getting hurt is something else than merely increased pain. By “things normal people do” the children most often thought of physical activities and school sports.

I thought about if I hurt myself physically, I mean in my body. If I, maybe run then fall and hit myself. (Child 4)

*Item 2. When I feel pain, I am afraid that something terrible will happen*.

The meaning of the word “terrible” was interpreted differently as illustrated by the citations.

Something that requires you to go to the emergency room. In other words, something that is actually wrong physically that is acute. (Child 1)

I think it would probably be that I would hurt myself more, much more. Something much worse is happening. That you get really sick. (Child 6)

The children mentioned that how they think and feel about their pain depends on what sort of pain they have, if it is familiar or something new. Five participants had received pain rehabilitation treatment and spontaneously described that they are not so afraid of the “regular” pain because that has been explained to them. However, they may be frightened if something new comes along that they do not recognize.

I'm more afraid when I get a new pain than when my persistent pain is maybe a little worse, mostly because then it's something new. (Child 1)

*Item 3. Pain causes my heart to beat fast or race*.

The item was understood as intended, namely that an emotional experience is included.

That I can get a little stressed by it at the same time as I can get a little sad about it. (Child 3)

After feed‐back from the lay‐panel, item 3 was shortened from “Pain causes my heart to beat fast and race” to just “Pain causes my heart to beat fast.” However, as the children who participated in the cognitive interviews spontaneously spoke about “heart racing” it was decided to change the wording back to the first version.

*Item 4. I cancel plans when I am in pain*.

The interviews showed a broad interpretation of what sort of plans that can be canceled. Most often children thought of plans of doing any activity, such as exercise or meet with friends.

I thought about playing tennis, meeting friends, exercising. (Child 6)

*Item 5. Feelings of pain are scary for me*.

The children had no difficulties understanding this item and it made sense to them why this was asked. Yet, they used “get afraid” or “feel afraid” instead of “scary” when they talked about the content of this item. This was discussed in the research group, and we decided to change the wording to make it more understandable and in line with how the Swedish language is used.

Some may feel a little scared when the pain just comes or if the pain has been there for quite a long time and you are afraid that it will not disappear or something else. (Child 2)

*Item 6. I worry when I am in pain*.

All six children had experience from worrying about their pain. Their worries related to the pain experience itself but also worries about not being able to do things the way one wants, such as concentrating in school. Worrying could apply to here and now but also relate to the future. Worrying and feeling afraid were used synonymously and to one child getting scared (as in item 5) and getting worried was the same. As a broad interpretation of worrying is desirable, no changes to this item were made.

Having pain is not something I'm that terribly afraid of. Because I have it regardless of what I want and what I don't. I'm not afraid of getting more pain or worried that it won't stop or anything. It's more how it affects everything else. (Child 1)

Worried and scared. It's the same question. Like, the same thing in my opinion. Because when you're worried, you're also a little scared, right? (Child 5)

*Item 7. I avoid making plans because of my pain*.

Referring to pain as “my pain” was accepted and agreed by the children. Planning was interpreted as one or at most a few weeks into the future. This applied regardless of the age of the children.

Yes, because the pain basically owns me. It is, it applies to me, it is mine, I own it. The pain is mine, it's a part of me. It's kind of me. It doesn't escape me. (Child 1)

I don't plan that far into the future. I think that now is now and this is how it is right now. Maybe a week or so into the future. (Child 2)

*Item 8. I put things off because of my pain*.

All children clearly understood the meaning of “put things off.”

If you're going to do something that day but you decide you want to do it the next day instead or so. (Child 4)

*Item 9. I stop any activity if I start to hurt or my pain becomes worse*.

Children recalled activities both in school and in their leisure time when responding to this item. Pain intensity and the form of activity were factors that influenced whether they stopped the activity or not.

I thought that if I get pain or get more pain I stop. (Child 4)

It depends on what things I do. Am I just in my room or watching a movie, then I wouldn't say that I'm stopping. But definitely if I play tennis or something. (Child 6)

*Item 10. I do not go to school because it makes my pain worse*.

For the children it was a fact that school makes pain worse. Still, that did not mean that they did not go to school. This led to some confusion when responding to this item. Yet, we did not perceive that any of the children answered at the wrong end of the scale.

The pain gets much worse. But I'll go there anyway. (Child 1)

I don't want to go to school, because it will make me hurt a lot more, but I guess it's also because I'm in a lot of pain right now. I don't want to go there because I'm in a lot of pain and I don't want it to get worse. But I've never, never been allowed not to go to school for that reason. (Child 5)

We also had concerns how “going to school” was interpreted, if children thought that it meant going from home to school or attending school. In fact, all of them thought that it meant attending school, which is the intention of the item.

#### 3.3.4 Relevance and Usability

The children recognized themselves in all 10 items and found them to be relevant for those who have had pain for a longer time. Overall, the FOPQC‐SF was perceived as useful and, except for one child who found item 10 stressful, the questionnaire did not upset the children.

It's probably number 10. That can be a bit difficult maybe. Because school absence, it's not fun to talk about when you're the person it's about. I don't think it's a bad thing to talk about, but it can be hard. It is hard because it feels like this is not normal. (Child 1)

The children also thought the questionnaire was useful in healthcare, for healthcare providers to understand each child and be able to help in a good way, but also as a way for children to express how they feel.

If you are in pain, a lot of pain, then they can understand you better if you answer this. Then they know more and can help in a better way. (Child 2)

Some children believed that it could have been emotionally difficult to answer the FOPQC‐SF at an early stage when they were more worried about their pain. Overall, though, it was considered by all participants that the questionnaire should be used also at an early stage.

Well, then it would probably have been a bit harder, I think. Because then you haven't realized why things have turned out the way they have. That you've been hurt and this whole part and it was a lot mentally. So, it would have been harder to answer that then. (Child 3)

Children who had participated in pain rehabilitation spontaneously commented that they would have given answers at the other end of the response scale before the treatment.

It would probably have been very different, that some numbers would probably have been much higher than they are now. (Child 2)

## 4 Discussion

In this study, a Swedish version of the FOPQC‐SF was translated, adapted, and co‐created in collaboration with the target group using the dual‐panel method combined with cognitive interviewing. Based on the interviews with the children, minor revisions were made regarding instructions, response options, item wording, and layout. The translation and adaptation process resulted in a Swedish version of the FOPQC‐SF that was found acceptable and relevant by the children. Also, the method used for translation and linguistic and contextual adaptation (i.e., dual‐panel method) was perceived by the researchers as easy to use and much more comprehensive compared to a forward‐backward translation.

### 4.1 Response Options

All children were able to understand and respond to the five‐point Likert Scale, which is in line with previous studies [23, 24, 25]. Still, difficulties responding to the middle response option were observed. The difficulty to understand and respond to the middle point of a five‐point scale, as well as an unwillingness to choose this option, has also been shown in previous studies [23, 26]. In our study, the middle response option “unsure” was found problematic by all children, and we therefore decided to only retain verbal definitions for the two extreme options. This alternative has previously been suggested by Perneger and co‐authors [27]. Future studies are needed to further explore how children relate to the response scale and to search for the most valid alternative.

### 4.2 Interpretation of the Wording

Generally, the children interpreted the wordings as intended, even when the expression was symbolic as for example in “put things off”. Some words meant different things to the children, for example “get hurt” and “activities.” This is not problematic since a wide and individual interpretation is wanted.

### 4.3 Reflections on the Methodology

#### 4.3.1 Dual‐Panel Method

The dual‐panel method was found to be an easy‐to‐use method for translating and adapting the FOPQC‐SF, allowing for the theoretical ground and intention of the questionnaire to be maintained. Although the children in the lay panel did not suffer from persistent pain, they contributed valuable insights on how wordings are interpreted and helped us to simplify the language and ensure that there were no misunderstandings. The children had constructive suggestions on how to remove superfluous words and improve the layout to make the questionnaire easy to read and answer. The dual‐panel process resulted in a well‐prepared first Swedish version of the FOPQC‐SF to be used for cognitive interviewing. An additional benefit was that this interview added to the content and face validity of the FOPQC‐SF.

#### 4.3.2 Cognitive Interviewing

Cognitive interviewing contributes important information about the development and evaluation of patient reported outcome measures (PROMS), but there is no definite consensus on how to use the method [20, 28]. Wright and colleagues [20] stated that a strict methodological protocol for cognitive interviewing could limit the applicability and scope of the method. Given that there is no standardized method for conducting cognitive interviewing, finding ways to use the method for this study was multifaceted.

In a systematic review, aiming to assess the quality of methodology reporting of studies using cognitive interviewing in the development of PROMs, 19 articles were included [20]. Six of these reported an unspecific qualitative analysis and the remaining 13 articles used seven different theoretical models or approaches in the analysis, such as thematic analysis and content analysis. Our analysis should be considered unspecific as it is mainly descriptive and not related to a theoretical model.

Due to the high degree of variability in how cognitive interviewing is used, the reporting should be comprehensive and systematic. Therefore, we used the Cognitive Interviewing Reporting Framework (CIRF) [20, 22] as a guide to structure the written report. We found CIRF to be useful as it helped us to be as thorough as possible in reporting the different parts of the study and to be transparent about how we used the method and analyzed the interviews. Using the reporting framework also made us aware of potential methodological weaknesses and need for improvement in future studies.

It could be argued that there were few participants in the cognitive interviewing. However, there is no definitive agreement on the number of participants to be included; in the past, 5 to 15 have been found to be sufficient [21]. Meadows [19] defines two approaches for determining the number of cognitive interviews to be performed: “do what you can” and “the Classical qualitative approach”, the latter meaning that interviews are conducted until saturation or no new results are obtained. Meadows also discussed that the most appropriate sample size is dependent on the aim of the study and that the likelihood of observing a given problem increases with an increased sample size. Our sample size of six children can be described following both the defined approaches, since all six children reported similar difficulties and opinions. Still, we cannot exclude that adding more participants would not have given new information. From an ethical point of view, we strived for minimizing the number of participating children needed to reach the main purpose of the study, namely to create a Swedish version of the FOPQC‐SF that was understood, relevant and usable for children with persistent pain.

### 4.4 Strengths and Limitations

The overarching strength of this study is that the Swedish version of the FOPQC‐SF was co‐created with the target group (i.e., children and adolescents). Important insights and information were gained through the interviews with the children, with and without persistent pain, that would have been missed using a plain forward‐backward translation. To increase our understanding of how children formulate answers the involvement of children, by using cognitive interviewing, is in line with the recommendations for the development of Patient Report Outcome Measures (PROMs) [26].

Five children who participated in the cognitive interviewing had undergone pain rehabilitation based on Acceptance and Commitment Therapy (ACT), which might have influenced their understanding of the questionnaire. However, these children demonstrated the ability to reflect on how their responses would have been different before rehabilitation. The experience of pain rehabilitation might also have improved their understanding of the mechanisms of persistent pain, including the concept of pain‐related fear and, thereby, the ability to reflect on what is relevant to ask about in a self‐report questionnaire.

## 5 Conclusion

The combination of the dual‐panel method and cognitive interviewing ensured the linguistic and contextual accuracy, as well as the target group's correct interpretation and understanding of the questionnaire. The rigorous process also provided evidence for content and face validity. We consider the Swedish version of the FOPQC‐SF a relevant and useful tool to be used in research as well as in clinical practice. Psychometric testing will provide further information about its clinical usefulness.

## Author Contributions

M.L. planned the study, collected, managed and analyzed data and was responsible for writing the draft of the manuscript. J.L. supervised the study. All authors contributed to the conceptualisation of the study, made important contribution to the analysis of data, revised the manuscript critically and approved the final manuscript.

## Conflicts of Interest

The authors declare no conflicts of interest.

## Supporting information


Data S1.

## Data Availability

The data that support the findings of this study are available on request from the corresponding author. The data are not publicly available due to privacy or ethical restrictions.
