# Supplementary material for: Questionnaire Translation, Adaptation, and Co‐Creation of the Swedish Version of the Fear of Pain Questionnaire Child Report Short Form
Source: Paediatr Neonatal Pain. 2025 Jul 4;7(3):e70011. doi: 10.1002/pne2.70011 (PMC12227015; doi:10.1002/pne2.70011)
Supplement: Supplementary file 1 — Data S1. [file PNE2-7-e70011-s001.docx]

**APPENDIX 1***.* English version of the FOPQC-SF.

**APPENDIX 2.** Overview of the cognitive interview procedure with examples of asked questions (probes).

**APPENDIX 3.** Swedish version of the FOPQC-SF.

**These questions ask about how you look at pain *when you hurt or are in pain for a few hours or days.***

**FOPQ-Child Report Short Form**

**Please read each statement carefully. Indicate how much you agree or disagree with each statement.**

|  | **Strongly**  **Disagree**  **0** | **Disagree**  **1** | **Unsure**  **2** | **Agree**  **3** | **Strongly**  **Agree**  **4** |
| --- | --- | --- | --- | --- | --- |
| **1. I can’t do all the things normal people do**  **because it’s so easy to hurt my body.** | 0 | 1 | 2 | 3 | 4 |
| **2. When I feel pain, I am afraid that something terrible will happen.** | 0 | 1 | 2 | 3 | 4 |
| **3. Pain causes my heart to beat fast or race.** | 0 | 1 | 2 | 3 | 4 |
| **4. I cancel plans when I am in pain.** | 0 | 1 | 2 | 3 | 4 |
| **5. Feelings of pain are scary for me.** | 0 | 1 | 2 | 3 | 4 |
| **6. I worry when I am in pain.** | 0 | 1 | 2 | 3 | 4 |
| **7. I avoid making plans because of my pain.** | 0 | 1 | 2 | 3 | 4 |
| **8. I put things off because of my pain.** | 0 | 1 | 2 | 3 | 4 |
| **9. I stop any activity if I start to hurt or my pain becomes worse.** | 0 | 1 | 2 | 3 | 4 |
| **10. I do not go to school because it makes my pain worse.** | 0 | 1 | 2 | 3 | 4 |

**References:**

1. L. C. Heathcote, R. P. Bhandari, I. Timmers, L. E. Harrison and L. E. Simons. Rapid identification and clinical indices of fear-avoidance in youth with chronic pain. Pain 2020:161:565-573
2. <https://bpp.stanford.edu/wp-content/uploads/2020/03/fopqc-sf.pdf>

***Appendix 1***

| **PART OF THE QUESTIONNAIRE** | | **INSTRUCTIONS FOR THE CHILD/ADOLESCENT** | | **PROBES** | |
| --- | --- | --- | --- | --- | --- |
| **Instructions** | *Could you please read the instruction at the top of the page and tell me in your own words what the instruction tells you to do.* | | *What do you think of the instructions?*  *What does "a few hours or days" mean to you?* | | |
| **Response options** | *Now you can read through the response options.* | | *Are the response options easy or difficult to understand?*  *What does* "unsure*" mean to you?*  *Do you think it will be easy or difficult to choose answer options?* | | |
| **Item 1-10** | | | **General probes** | | **Specific item probes** |
|  | *Now you're going to answer the questionnaire from beginning to end and then we are going to talk a little more about each question.*  *Try to answer the questions as best as you can at your own pace and if you have any problems or if something is difficult to understand, ask me for help.*  *Feel free to tell me what you find difficult and we will go back to this when you are done.*  *Try to think aloud while reading and answering the questions.* | | *I saw that you hesitated when answering: Can you tell me what you were thinking? (comprehension)*  *Can you repeat the question in your own words? (language)*  *Do you think this question is important for someone who has pain? Why/why not? (relevance)*  *How did you choose this particular response option?* | | Item 2: *What could "something terrible" be?*  Item 3: *What does “my heart beats fast or race” mean to you?*  Item 4: *“I don't do things I was supposed to do when I'm in pain.” What are things?*  Item 7: *How far ahead were you thinking when you answered?*  Item 10: *Is there a difference between “hurt” and “feeling pain”?* |
| **Finishing questions** | *Now we have gone through the entire questionnaire and will soon be ready. I will just ask a few more questions.* | | *What did you think of the questionnaire?*  *Did you find the questions meaningful to you? If yes: In what way? If no: Why?*  *Was there any question that made you sad or upset. If yes: Which question?*  *Do you think this questionnaire would be good to use in healthcare? If yes: In what way? If no: Why?* | | |

***Appendix 2***

**Dessa påståenden handlar om hur du** **har det** **när du har ont eller känner smärta ofta eller en längre tid.**

**SMÄRTRELATERAD RÄDSLA – FRÅGEFORMULÄR FÖR BARN OCH UNGDOMAR**

**FEAR OF PAIN QUESTIONNAIRE – CHILD REPORT SHORT FORM**

**Läs varje mening noga. Ringa in det svar som stämmer bäst in på dig.**

| Datum  Personnummer  Namn | Stämmer inte alls  0 | 1 | 2 | 3 | Stämmer helt  4 |
| --- | --- | --- | --- | --- | --- |
| 1. Jag kan inte göra allt som andra gör eftersom jag har lätt för att skada mig. | 0 | 1 | 2 | 3 | 4 |
| 2. När jag känner smärta är jag rädd att något hemskt ska hända mig. | 0 | 1 | 2 | 3 | 4 |
| 3. Smärtan gör att mitt hjärta slår snabbare eller dunkar hårt. | 0 | 1 | 2 | 3 | 4 |
| 4. Jag gör inte saker jag hade tänkt göra när jag har ont. | 0 | 1 | 2 | 3 | 4 |
| 5. Att känna smärta gör mig rädd | 0 | 1 | 2 | 3 | 4 |
| 6. Jag blir orolig när jag har ont. | 0 | 1 | 2 | 3 | 4 |
| 7. Jag planerar inte saker på grund av min smärta. | 0 | 1 | 2 | 3 | 4 |
| 8. Jag skjuter upp saker på grund av min smärta. | 0 | 1 | 2 | 3 | 4 |
| 9. Jag slutar med det jag håller på med om jag får ont eller om min smärta blir värre. | 0 | 1 | 2 | 3 | 4 |
| 10. Jag går inte till skolan eftersom det gör att min smärta blir värre. | 0 | 1 | 2 | 3 | 4 |

**Referens:** L. C. Heathcote, R. P. Bhandari, I. Timmers, L. E. Harrison and L. E. Simons. Rapid identification and clinical indices of fear-avoidance in youth with chronic pain. Pain 2020:161:565-573

Svensk översättning och anpassning till svenska förhållanden©

Malin Lanzinger, doktorand, leg fysioterapeut, Jan Lexell, professor, överläkare, Marcelo Rivano Fischer, docent, leg psykolog

och Sophie Jörgensen, dr med vet, specialistläkare, Lunds universitet, Skånes universitetssjukhus och Ängelholms sjukhus.

Om du vill använda frågeformuläret kontakta [malin.lanzinger@med.lu.se](mailto:malin.lanzinger@med.lu.se)

***Appendix 3***
